# Supplementary material for: Should I stay or should I go—Medical assistants´ experiences and coping with patient demand and lack of appreciation during the Covid-19 pandemic
Source: PLoS One. 2025 Apr 17;20(4):e0320953. doi: 10.1371/journal.pone.0320953 (PMC12005545; doi:10.1371/journal.pone.0320953)
Supplement: S1 File — (DOCX) [file pone.0320953.s001.docx]

| **Topic I: Introduction** | |
| --- | --- |
| Thank you very much for agreeing to take part in this interview.  My name is (...), I am a research associate at the Institute of General Practice and Family Medicine at the Ruhr University Bochum.  We would like to learn more about pandemic management over the past year in our interview with you. For example, how your day-to-day work has changed and what challenges you have faced and possibly solved so far (and if so, how?). These are questions about your personal experiences. Your opinions and subjective perceptions are therefore very important to us and we would like to ask you to be as open as possible about them.  To begin with, I would like to say a few words about the interview process. First of all, we would like to record the interview so that we can concentrate better on your case and take fewer notes. We will, of course, treat the recording confidentially. It will of course be completely pseudonymized, so that no conclusions can be drawn about your person after the recording. The file will be deleted after recording. Do you agree to the recording? [If yes, switch on the audio recorder now - if no, take notes]. The audio recorder is now turned on.  Your participation in this interview is, of course, voluntary and you can stop the interview at any time or refuse to answer individual questions. Nevertheless, I would ask you to answer as many questions as possible in the interview so that I can better comprehend everything you have said.  Do you have any questions about the process? Otherwise we can start now. | Obtain consent for recording  Pseudonymization  *[Turn on the audio recorder if necessary].*  Voluntariness |

| **Topic II: Comparison of situations** | |
| --- | --- |
| Let's start by simply telling me about your current day-to-day work. What is it like at the moment?  How are vaccinations organized in your practice? | - - Focus on: Lockdown, equipment (masks, gloves), patient interaction, vaccinations (organization and provision of vaccine) - - Are vaccination appointments attended? How do patients react to unwanted vaccines (like AstraZeneca)? Are sufficient vaccine doses available? - - Narrative conversation starter to build on the following questions |
| How does your everyday working life currently differ from your usual working life outside of pandemic times? | - - Comparison of current everyday life with “normal” everyday life - - What has changed? |

| **Topic III: Pandemic development** | |
| --- | --- |
| In this section, I would like to ask you to remember back to a year ago, if possible: How did you feel at the beginning of the pandemic? | - Professionally, personally - what do you particularly remember? |
| What was your everyday working life like at the beginning of the pandemic?  Can you tell us about a particular day during that time? | - - What (sudden) changes have occurred? - - Changes: Cooperation with colleagues, communication, cooperation with other stakeholders |
| How has your everyday working life changed during the pandemic? | - With regard to lockdown easing, renewed lockdown |
| How has the pandemic affected the profitability of your practice? | - What is the financial situation of GP practices? |

| **Topic IV: Support** | |
| --- | --- |
| Have you been provided with any materials during the pandemic? And to what extent? | - Face masks, gloves, overalls -> enough material or did disposable items have to be used several times? |
| Which support would you have wished for in your everyday work? By whom or from which area? | - Politics, Medical Association, Association of Statutory Health Insurance Physicians |
| What digital aids would you have needed for your day-to-day work?  Or: Was it possible for you to offer a video consultation? | - How successful was this digital consultation? Was it well received? Was it used often? |

| **Topic V: Prospects** | |
| --- | --- |
| What specific measures should be introduced in GP practices to prevent the further spread of the coronavirus? | - Are there still any measures that can / should be taken? |
| What conclusions have you drawn from the Covid-19 pandemic?  What could have been done differently?  What would you like to change in the future?  Have you taken measures to possibly prepare for another pandemic? If so, which ones? | - What could / should have been done differently? - What would you like to happen in the future? |

| **Topic VI: Conclusion** | |
| --- | --- |
| We have now asked all the questions that are important to us. Is there anything else we haven't discussed so far that you would like to add? |  |

| **Conclusion & thanks**  *At this point, we would like to switch off the recording device [switch off recording device]. We thank you for your time and your willingness to participate in our study.*  If you have any questions, you can contact the person listed on the project description at any time by phone or e-mail. |
| --- |

| **Postscript (for interviewer only)**  **After completing the interview, please write down/dictate your impressions and comments on the following points:**  Where did the interview take place?  Were there any disruptions during the interview? If yes, which ones? Were there any other special features?  Which topics/questions did you leave out and why?  What was the development dynamic of the entire interview?  What was the mood and behavior of the interviewee?  How did you feel during the interview?  How would you rate the relationship between you and the interviewee?  After the interview was completed, did the interviewee provide any important information that was not recorded? If so, what information? |
| --- |
