# Supplementary material for: Should I stay or should I go—Medical assistants´ experiences and coping with patient demand and lack of appreciation during the Covid-19 pandemic
Source: PLoS One. 2025 Apr 17;20(4):e0320953. doi: 10.1371/journal.pone.0320953 (PMC12005545; doi:10.1371/journal.pone.0320953)
Supplement: S2 File — (DOCX) [file pone.0320953.s002.docx]

List of codes

| **List of codes** | **Frequency** |
| --- | --- |
| Codesystem | 1413 |
| Consent and introduction | 21 |
| Pandemic development | 10 |
| Changes during the pandemic | 15 |
| Comparison of work without a pandemic | 11 |
| Beginning of the pandemic | 13 |
| Personal perception | 18 |
| Structural changes at the beginning | 25 |
| Infection consultation and swabs | 31 |
| Challenges at the beginning | 34 |
| Solutions for the initial challenges | 19 |
| Patient interaction at the beginning of the pandemic | 15 |
| Changed work structures and organization | 20 |
| Additional work? | 69 |
| Corona restrictions | 20 |
| Digitalization | 31 |
| Teamwork | 8 |
| Distribution of roles & tasks | 33 |
| Atmosphere and cooperation | 29 |
| Lack of information and transparency + Bureaucracy | 52 |
| Wishes at the beginning | 14 |
| Support programs | 18 |
| Personnel support | 5 |
| Social support | 3 |
| Financial support | 7 |
| Financial losses / short-time working | 33 |
| Material support | 22 |
| Shortage of materials | 24 |
| Overtime compensation? | 5 |
| Summer liberalization measures | 10 |
| Challenges during the liberalizations | 3 |
| Solutions during the liberalizations | 0 |
| Patient interaction during the liberalizations | 3 |
| Wishes in summer | 0 |
| Third wave and renewed lockdown | 3 |
| Challenges during the 3rd wave | 39 |
| Solutions during the 3rd wave | 13 |
| Patient interaction in the 3rd wave + vaccinations | 34 |
| Wishes during the 3rd wave | 7 |
| Corona vaccinations | 18 |
| Organization of vaccinations in the practice | 46 |
| Dealing with different vaccines | 31 |
| Supply of vaccine | 38 |
| General challenges | 10 |
| Social and societal responsibility | 12 |
| Patient care | 7 |
| Difficult patient contact | 46 |
| Patient interaction | 41 |
| Information work | 28 |
| Compliance with hygiene & protective measures | 26 |
| Treatment restrictions | 37 |
| Physical and mental stress | 32 |
| Work overload | 24 |
| Worries about infection | 10 |
| Existential worries | 2 |
| Effects of isolation | 7 |
| Stress caused by protective equipment | 14 |
| Attitude towards one's own profession | 19 |
| Resilience | 23 |
| Motivation for the working day | 6 |
| Values for vaccination / protective measures | 27 |
| Basic trust in politics / state | 3 |
| Changes in the social environment | 4 |
| Effects on private life | 23 |
| Restrictions in everyday private life | 7 |
| Prospects | 0 |
| What could have been done differently? | 10 |
| Politically | 17 |
| Medial | 15 |
| Wishes for the future | 27 |
| Potential containment measures for GP practices | 17 |
| Future pandemics | 21 |
| Reference to the pandemic plan | 1 |
| Interesting / New | 14 |
| Criticism of the responsible health minister | 13 |
| Closure | 20 |
